# Nutrition Literacy of Middle School Students and Its Influencing Factors: A Cross-Sectional Study in Chongqing, China

**DOI:** 10.3389/fpubh.2022.807526

**Published:** 2022-03-15

**Authors:** Mao Zeng, Yuzhao Zhu, Zhengjie Cai, Jinli Xian, Shengping Li, Tiankun Wang, Zumin Shi, Manoj Sharma, Yong Zhao

**Affiliations:** ^1^Department of Nutrition and Food Hygiene, School of Public Health and Management, Chongqing Medical University, Chongqing, China; ^2^Research Center for Medicine and Social Development, Department of Nutrition and Food Hygiene, School of Public Health and Management, Chongqing Medical University, Chongqing, China; ^3^The Innovation Center for Social Risk Governance in Health, Department of Nutrition and Food Hygiene, School of Public Health and Management, Chongqing Medical University, Chongqing, China; ^4^Department of Communicable Disease Control and Prevention, Chengdu Shuangliu District Disease Prevention and Control Center, Chengdu, China; ^5^Department of Health Behavior and Social Medicine, West China School of Public Health and West China Fourth Hospital, Sichuan University, Chengdu, China; ^6^Department of Clinical Nutrition, West China Hospital of Sichuan University, Chengdu, China; ^7^Human Nutrition Department, College of Health Sciences, QU Health, Qatar University, Doha, Qatar; ^8^Department of Social and Behavioral Health, School of Public Health, University of Nevada, Las Vegas, NV, United States; ^9^Chongqing Key Laboratory of Child Nutrition and Health, Children's Hospital of Chongqing Medical University, Chongqing, China

**Keywords:** nutrition literacy, nutrition, middle school students, Chongqing, influencing factors

## Abstract

Nutrition literacy plays an important role in children's dietary habits and nutrition. This study aimed to analyse the status of nutrition literacy and its influencing factors amongst middle school students in Chongqing, China. “Nutrition literacy scale for middle school students in Chongqing” was used in 29 districts of Chongqing in September 2020. The scores of nutrition literacy and its' three sub-domains (functional, interactive and critical nutrition literacy) were divided into low and high groups based on their median scores. Binary logistic regression was used to measure the influencing factors of nutrition literacy. A total of 18,660 middle school students were included in this study. The median of nutrition literacy of middle school students was 61.68 (IQR = 14.37). Interactive nutrition literacy had the highest score (median = 70.00, IQR = 20.00), followed by functional nutrition literacy (median = 68.69, IQR = 14.14) and critical nutrition literacy (median = 45.83, IQR = 25.00). Students who were the minority (OR = 0.71, 95% CI = 0.637–0.785), in senior high school (OR = 0.51, 95% CI = 0.477–0.548), in rural areas (OR = 0.85, 95% CI = 0.790–0.911), receiving school meal support from the government (OR = 0.63, 95% CI = 0.591–0.664), with other caregivers' parenting (OR = 0.86, 95% CI = 0.805–0.914), with parents having a low level of education and with an abnormal BMI [thin (OR = 0.91, 95% CI = 0.837–0.990), overweight (OR = 0.87, 95% CI = 0.785–0.968), and obese (OR = 0.83, 95% CI = 0.767–0.902)] presented less probability of being a high level of nutrition literacy. Our results could assist public health authorities in developing strategies of nutrition literacy promotion for references and theoretical foundations.

## Introduction

Childhood is a key stage exhibiting rapid changes in physical growth, psychosocial development and behavioral modifications (1). Unhealthy eating habits can predispose children to chronic disease (2) and weaken their learning capacity (3). The growth of children is seriously threatened by malnutrition caused by poor dietary quality (4). In addition, unhealthy eating behavior can result in exceeded diet (5). Adolescence is considered a vital period to lay the foundation for health, investment in adolescent health can promote adult health and the life quality of the next generation (6). Furthermore, some studies have demonstrated that the Coronavirus Disease 2019 (COVID-19) pandemic has brought unhealthy dietary effects to children and adolescents, such as the increased intake of potato chip, red meat and sugary beverage (7, 8).

Nutrition literacy (NL) is recognized as a key determinant of healthy dietary habits and nutritional status at the individual level (9), which can be defined as the capacity to obtain, process and understand nutrition information and the materials needed to make appropriate decisions regarding one's health (10). NL is comprehended as a “specific form of health literacy (11),” “similar to health literacy (10),” or “health literacy applied to the field of nutrition (12).” Krause et al. (13) has described three sections of NL as “functional, interactive and critical” nutrition literacy. Functional nutrition literacy (FNL) refers to the basic reading and writing skills necessary to understand and follow simple nutrition information. Interactive nutrition literacy (INL) refers to the cognitive and interpersonal communication skills required to receive nutrition information and communicate appropriately with nutrition consultants. Critical nutrition literacy (CNL) refers to the ability to critically analyse nutrition information, raise awareness about nutrition and participate in studies aimed at solving nutrition barriers (14, 15). On the basis of Food and Nutrition Literacy (FNLIT) questionnaire developed by Doustmohammadian et al. (16) and a systematic review of existing measurement tools of nutrition and food literacy (17), FNL, INL, and CNL are composed of “obtain, understand, apply, interact, emotional, discussion, analysis, critical skills and media literacy.”

Previous researches have proved that educational level, income, residence, age and gender are the main sociodemographic factors affecting NL. Education, income and urban residence are positively associated with NL (18–20). With the growth of age, the NL may be lower due to cognitive, visual and auditory impairment and less acquisition of nutrition and health information (21). In general, female has a higher level of NL than male (22). It is worth noting that guardian is a significant factor influencing children's NL, and children with parental guardians have higher NL than children who are cared for by one parent or others (23). Furthermore, media literacy (22) and nutrition education (24) play a significant role in enhancing NL level.

To date, some studies on NL amongst children and adolescents had been conducted. Nutrition literacy amongst young adults in Turkey was evaluated at an intermediate level (1). The result of a study in Iran indicated that more than 10% of students had a low total food and NL (16). In 2018, in Fengtai, Beijing, China, only 8.4% of primary school students and 6.4% of junior high school students had a comprehensive grasp of nutrition knowledge (25), and in 2019, the NL level of school-age children in Baoding, Hebei Province, was only 2.09% (26). Adequate NL requires an individual to comprehend nutrition concepts and to possess simple numeracy skills (27). Without these skills, there will be difficulties in understanding the concepts of a healthy diet, reading nutrition information, determining healthy food options and utilizing nutrition information sources. Promoting NL in children can help them develop healthy food habits and improve their health and nutritional status (28–30). Despite these initial findings, the number of studies on the NL and its influencing factors amongst students is limited, and no relevant research have been conducted amongst middle school students in Chongqing, China.

This study aimed to explore the NL status and its influencing factors amongst middle school students in Chongqing, China, which could pave the way for formulating strategies to improve the NL of Chinese students.

## Materials and Methods

### Participants and Procedure

This study was a cross-sectional study, conducted in 239 schools of 29 districts of Chongqing in September 2020. Online survey for data collection was completed by students namelessly and independently under the guidance of class teachers. All participants were informed about the study, and consents were obtained before filling in the scale anonymously. This study was approved by the Ethics Committee of Chongqing Medical University. A total of 21,084 middle school students were investigated, and 917 outliers and 1,507 missing data for responding “education of parents” with “don't know” were excluded. The final effective questionnaires were 18,660.

### Tools

Data were collected by the “Nutrition literacy scale for middle school students in Chongqing (CM-NLS).” “CM-NLS” was designed on the basis of the Dietary Guidelines for School-age Children in China 2016 (31), characteristics of middle school students in Chongqing (32) and other literature and materials (17, 33). Three experiments were conducted to develop CM-NLS: a theoretical framework and an initial item pool were established based on the literature review, two-rounds delphi method was used to explore the suitable items, and item evaluation and reduction were performed using the Classical test theory (CTT). In addition, the final scale was tested for its validity and reliability amongst 462 middle school students. Details of this approach have been described previously (34). “CM-NLS” consisted of two sections.

Section A obtained the demographic characteristics of respondents and the sources and difficulties in obtaining nutrition information. Self-reported data on demographic information were collected: gender (boy vs. girl), ethnicity (Han vs. minorities), grade (grade 7 and 8 students who were in junior high school, grade 10 and 11 students who were in senior high school), whether boarding in school (yes vs. no), residence (urban vs. rural), whether receiving school meal support from the government (yes vs. no), primary caregiver (parents vs. others) and the level of education of parents (primary schools and below, junior high school, senior high school/technical secondary school/vocational high school, college/bachelor degree or above). According to the International Obesity Task Force (IOTF), Body Mass Index (BMI, weight/height^2^) was categorized according to age and gender of children (35). BMI was divided into thin, normal, overweight and obese groups by classification criteria of 2–18 years old children. Sources and difficulties in obtaining nutrition information were measured by three questions: “how do you get nutrition information current?” “how will you get nutrition information in the future?” and “what difficulties do you have in obtaining nutrition information?” The response options for the first two questions were “newspapers and magazines/television and radio/network/parents or relatives or friends/the classroom/others.” And the response options for the last question included “need a lot of time to obtain information/difficult to understand the content/difficult to verify credibility/difficult to understand other languages/others.” The “other” option of the three questions were all open-ended answers.

Section B measured the NL of middle school students. This part consisted of three sub-domains (FNL, INL, and CNL) with 52 items. In this study, FNL consisted of 35 items with three skills, namely, “obtain skill,” “understand skill,” and “apply skill.” Interactive nutrition literacy consisted of 5 items with “interact skill.” Critical nutrition literacy consisted of 12 items with “media literacy” and “critical skill.” Please see the Supplementary Material “Section B of CM-NLS” for specific items of FNL/INL/CNL. A centesimal measure was used in calculating the sum score (0 to 100 points) of total and each sub-domain, with a higher score indicating better NL level. The scores of NL, FNL, INT, and CNL were divided into low and high levels based on their median scores (61.68, 68.69, 70.00, and 45.83, respectively).

Most of the participants completed this scale for ~15 min. Cronbach's coefficient of the “CM-NLS” was 0.902, and Cronbach's coefficient of three subscales was 0.804, 0.926, and 0.927, respectively. The KMO value of “CM-NLS” was 0.946, and the result of the Bartlett test of sphericity reached the level of significance (*P* < 0.001), indicating that the questionnaire had reasonable reliability and validity.

### Statistical Analysis

Data analysis was performed using STATA version 16 (STATA Corporation, College Station). Descriptive statistics was conducted to describe participants' characteristics and the sources and difficulties in obtaining nutrition information. χ^2^ tests were conducted to examine the distribution of NL scores. As the Intra Class Correlation (ICC) value was 0.01, we did not conduct multilevel analysis. Binary logistic regression was used to identify factors associated with NL.

## Results

### Participants' Characteristics

As shown in Table 1, amongst the 18,660 students, 50.16% were boys, 88.86% were Han ethnicity, 65.29% were boarding in school, 49.08% were receiving school meal support from the government, and 71.35% reported their parents as primary caregivers. Most of their parents' educational level was junior high school (father accounted for 51.12%, and mother accounted for 45.79%). Furthermore, 9.36% and 17.94% of the students were overweight and obese, respectively.

**Table 1:** Demographic characteristics of students in Chongqing (*N* = 18,865).

**Variables**		***n***	**%**
Year (mean, SD)		14.28, 1.75	
Gender	Boy	9,359	50.16
	Girl	9,301	49.84
Ethnicity	Han	16,581	88.86
	Minority	20,79	11.14
Grade	Junior high school	10,670	57.18
	Senior high school	7,990	42.82
Whether boarding in school	Yes	12,183	65.29
	No	6,477	34.71
Residence	Urban	9,062	48.56
	Rural	9,598	51.44
Whether receiving school meal support from the government	Yes	9,159	49.08
	No	9,501	50.92
Primary caregiver	Parents	13,313	71.35
	Others	5,347	28.65
Father's education	Primary schools and below	4,176	22.38
	Junior high school	9,539	51.12
	Senior high school/technical secondary school/vocational high school	3,264	17.49
	College/bachelor degree or above	1,681	9.01
Mother's education	Primary schools and below	8,555	45.85
	Junior high school	8,638	45.79
	Senior high school/technical secondary school/vocational high school	2,865	15.35
	College/bachelor degree or above	1,388	7.44
BMI (Kg/m^2^) (mean, SD)		22.38 (1.75)	
BMI group	Normal	10,579	56.69
	Thin	2,986	16.00
	Overweight	1,747	9.36
	Obese	3,348	17.94

### Methods and Difficulties in Obtaining Nutrition Information

A total of 835 (4.47%) middle school students in Chongqing had never received nutrition information. As shown in Table 2, nutrition information were primarily obtained from the internet, classroom, television and radio, and students hoped to obtain information through the above-mentioned channels in the future. A significant difference was found between boys and girls (*P* < 0.05). Many students encountered difficulties in obtaining information, such as “difficult to verify credibility (9,191, 49.26%)” and “difficult to understand content (7,557, 40.50%).” Compared with girls, a higher proportion of boys needed a lot of time to obtain information, had difficulty in understanding content and understanding other language; girls presented higher proportion of having difficult to verify credibility and facing other obstacles (*P* < 0.05).

**Table 2:** Channels and obstacles of nutrition information obtained by gender *N* (%).

**Channel**	**Total**	**Boy**	**Girl**	**χ^2^**	***P***
**Current** ^**a**^					
Newspapers and magazines	6,660 (35.69)	3,129 (46.98)	3,531 (53.02)	41.718	<0.001***
Television and radio	10,913 (58.48)	5,269 (48.28)	5,644 (51.72)	36.908	<0.001***
Network	12,295 (71.78)	5,901 (48.00)	6,394 (52.00)	68.287	<0.001***
Parents or relatives or friends	10,747 (57.59)	5,041 (46.92)	5,705 (53.08)	103.468	<0.001***
The classroom	11,720 (62.82)	5,630 (48.04)	6,090 (51.96)	56.538	<0.001***
Others	1,556 (8.33)	725 (46.59)	831 (53.41)	8.813	0.003**
**Future** ^**b**^					
Newspapers and magazines	9,062 (48.56)	4,241 (46.80)	4,821 (53.20)	79.352	<0.001***
Television and radio	11,690 (62.65)	5,715 (48.89)	5,975 (51.11)	20.111	<0.001***
network	13,544 (72.58)	6,684 (49.35)	6,860 (50.65)	12.810	<0.001***
Parents or relatives or friends	9,393 (50.34)	4,544 (48.38)	4,849 (51.62)	23.943	<0.001***
The classroom	13,479 (72.23)	6,594 (48.92)	6,885 (51.08)	29.612	<0.001***
Others	2,193 (11.75)	1,019 (46.47)	1,184 (53.53)	13.530	<0.001***
**Obstacles** ^**c**^					
Need a lot of time to obtain information	6,296 (33.74)	3,290 (52.26)	3,006 (47.74)	16.762	<0.001***
Difficult to understand content	7,557 (40.50)	3,831 (50.69)	3,726 (49.31)	1.478	0.024*
Difficult to verify credibility	9,191 (49.26)	4,458 (48.50)	4,733 (51.50)	19.759	<0.001***
Difficult to understand other language	6,367 (34.12)	3,330 (52.30)	3,037 (47.70)	17.796	<0.001***
Others	2,118 (11.35)	1,010 (47.69)	1,108 (52.31)	5.825	0.016*

*P <0.05, **P <0.01, ***P <0.001.

*^a^n = 6,660, excluded 835 participants had never received nutrition information.*

*^b^n = 9,062, included all participants who answered the questions.*

*^c^n = 6,296, included all participants who answered the questions*.

### Nutrition Literacy of Middle School Students

The median of NL of middle school students in Chongqing was 61.68 (IQR = 14.37, *n* of a high level = 8,972). INL had the highest score (median = 70.00, IQR = 20.00, *n* of a high level = 9,309), followed by FNL (median = 68.69, IQR = 14.14, *n* of a high level = 8,672) and CNL (median = 45.83, IQR = 25.00, n of a high level = 9,815). “Apply skill” had the highest score (median = 40.00, IQR = 8.00, n of a high level = 9,132), followed by “understand skill” (median = 20.00, IQR = 6.00, *n* of a high level = 8,566), “interact skill” (median = 14.00, IQR = 4.00, n of a high level = 9,309), “media literacy” (median = 13.00, IQR = 10.00, *n* of a high level = 9,220), “obtain skill” (median = 8.00, IQR = 3.00, *n* of a high level = 7,423), and “critical skill” (median = 8.00, IQR = 3.00, *n* of a high level = 7,836).

### Influencing Factors of Nutrition Literacy

Binary logistic regression was utilized to confirm the influencing factors of NL and three sub-domains (Table 3). A low possibility of a high level of NL was observed for the following students: students who were the minority (OR = 0.71, 95% CI = 0.637–0.785), in senior high school (OR = 0.51, 95% CI = 0.477–0.548), in rural areas (OR = 0.85, 95% CI = 0.790–0.911), receiving school meal support from the government (OR = 0.63, 95% CI = 0.591–0.664), with other caregivers' parenting (OR = 0.86, 95% CI = 0.805–0.914), with parents having a low level of education and with an abnormal BMI [thin (OR = 0.91, 95% CI = 0.837–0.990), overweight (OR = 0.87, 95% CI = 0.785–0.968) and obese (OR = 0.83, 95% CI = 0.767–0.902)] compared with those who were Han, in junior high school, in urban areas, not receiving school meal support, with parents' parenting, with parents having a high level of education and with normal BMI.

**Table 3:** Binary logistic regression for nutrition literacy of participants.

**Independent variable**	**NL**		**FNL**		**INL**		**CNL**	
	**OR**	**95% CI**	**OR**	**95% CI**	**OR**	**95% CI**	**OR**	**95% CI**
**Gender** (Girl)	Refer.							
Boy	0.95	0.896–1.005	0.88	0.830–0.931***	0.98	0.924–1.037	1.02	0.962–1.079
**Ethnicity** (Han)	Refer.							
Minority	0.71	0.637–0.785***	0.67	0.601–0.749***	0.77	0.695–0.849***	0.90	0.820–0.996*
**Grade** (Junior high school)	Refer.							
Senior high school	0.51	0.477–0.548***	0.42	0.387–0.445***	0.51	0.475–0.545***	0.967	0.905–1.036
**Whether boarding in school** (No)	Refer.							
Yes	0.95	0.881–1.018	0.87	0.806–0.933***	1.03	0.963–1.112	1.05	0.977–1.126
**Residence** (Urban)	Refer.							
Rural	0.85	0.790–0.911***	0.83	0.768–0.888***	0.99	0.920–1.058	0.91	0.848–0.974**
**Whether receiving school meal support from the government** (No)	Refer.							
Yes	0.63	0.591–0.664***	0.54	0.510–0.573***	0.90	0.849–0.952***	0.88	0.829–0.931***
**Primary caregiver** (Parents)	Refer.							
Others	0.86	0.805–0.914***	0.90	0.841–0.955*	0.94	0.884–1.003	0.93	0.873–0.992*
**Father's education** (Primary schools and below)	Refer.							
Junior high school	1.11	1.025–1.211*	1.12	1.031–1.224**	1.14	1.050–1.237**	1.06	0.976–1.147
Senior high school/technical secondary school/vocationaHigh school	1.22	1.089–1.369**	1.20	1.070–1.351**	1.04	0.930–1.165	1.10	0.985–1.232
College/bachelor degree or above	1.45	1.232–1.710***	1.56	1.317–1.840***	1.08	0.920–1.269	1.18	1.006–1.385*
**Mother's education** (Primary schools and below)	Refer.							
Junior high school	1.25	1.157–1.351***	1.25	1.157–1.356***	1.15	1.067–1.243***	1.08	1.002–1.164*
Senior high school/technical secondary school/vocational High school	1.30	1.157–1.458***	1.32	1.173–1.484***	1.20	1.066–1.341**	1.10	0.983–1.232
College/bachelor degree or above	1.45	1.232–1.710***	1.56	1.317–1.840***	1.08	0.920–1.269	1.18	1.006–1.385*
**BMI** (Normal)	Refer.							
Thin	0.91	0.837–0.990*	0.93	0.857–1.017	0.96	0.887–1.047	0.95	0.873–1.027
Overweight	0.87	0.785–0.968*	0.81	0.724–0.897***	0.95	0.855–1.053	0.95	0.860–1.056
Obese	0.83	0.767–0.902***	0.75	0.687–0.811***	0.95	0.873–1.023	1.01	0.935–1.093

*P <0.05, **P <0.01, ***P <0.001.

For the three sub-domains, students who were boys (OR = 0.88, 95% CI = 0.830–0.931), the minority (OR = 0.67, 95% CI = 0.601–0.749), in senior high school (OR = 0.42, 95% CI = 0.387–0.445), boarding in school (OR = 0.87, 95% CI = 0.806–0.933), in rural areas (OR = 0.83, 95% CI = 0.768–0.888), receiving school meal support (OR = 0.54, 95% CI = 0.510–0.573), with other caregivers' parenting (OR = 0.90, 95% CI = 0.841–0.955) and overweight (OR = 0.81, 95% CI = 0.724–0.897), obese students (OR = 0.75, 95% CI = 0.687–0.811) presented less probability of being a high level of FNL compared with students who were girls, Han, in junior high school, not boarding, in urban areas, not receiving school meal support, with parents' parenting and normal BMI students. Students who were the minority (OR = 0.77, 95% CI = 0.695–0.849), in senior high school (OR = 0.51, 95% CI = 0.475–0.545), and receiving school meal support (OR = 0.90, 95% CI = 0.849–0.952) were less likely to report a high level of INL compared to students who were Han, in junior high school, and not receiving school meal support. The minority (OR = 0.90, 95% CI = 0.820–0.996), rural (OR = 0.91, 95% CI = 0.848–0.974), receiving school meal support (OR = 0.88, 95% CI = 0.829–0.931), and with other caregivers' parenting (OR = 0.93, 95% CI = 0.873–0.992) students were less likely to report a high level of CNL than Han, urban, not receiving school meal support, and with parents' parenting students.

## Discussion

Nutrition literacy can affect individuals' dietary behavior in a positive manner (1). Thus, considerable attention should be given to NL during the COVID-19 epidemic (36). Our findings showed that the NL of middle school students in Chongqing was at the moderate level. Gender, ethnicity, grade, BMI, boarding, residence, school meal support from the government, primary caregiver and the level of education of parents were the predictors of NL amongst middle school students, which can be divided into the following aspects: individual, school environment and family (37).

The present study demonstrated that the NL of middle school students in Chongqing was at the moderate level and showed a comparable result to Liu et al.'s study conducted amongst participants in grades 3–8 (38). In Liu et al.'s (26) research, the CNL score was the highest, followed by FNL and INL, and in our study, the rate of a high level of FNL was the lowest. As childhood is a transitory phase with strong learning ability and high malleability, more nutrition education should be provided for middle school students in the absence of nutrition knowledge, and advanced information technologies could be applied to make nutrition information more accessible (39). With regard to the difficulties in obtaining nutrition information, methods to make students understand food labels more easily should be explored, such as popularizing specific label elements and terminologies and simplifying or translating food labels (40). Although students obtained nutrition information primarily by the internet, television and radio, these tools can be used to bolster their media nutrition literacy and CNL. In addition, considering the negative influence of unhealthy food advertising (41), students should be restricted to exposure to unhealthy food promotions, particularly advertising of high-fat, salty or sugary foods (42). With regard to the classroom approach, only providing nutrition knowledge at school was not sufficient; therefore, farm-to-school program, school gardens and cooking programs that incorporate different nutrition information should increase in the future (43).

Similar to Kalkan (1), this study found that girls were more likely to report a high level of FNL than boys. Girls typically paid more attention to their dietary intake and more likely acquired nutrition knowledge. In addition, in Chinese tradition, women were usually engaged in cooking and selecting food for family members. Consistent with prior research (44, 45), the NL level differed in grade and ethnicity. Different from other studies (18, 46), senior high school students were less likely to be a high level of NL/FNL/INL than junior high school students in this study probably because nutrition-related courses were rarely conducted in senior high school for great enrolment pressure (39). Moreover, family members of senior high school students usually focus more on students' study, resulting in insufficient nutrition education at home. Considering the academic pressure, senior high school students also lacked of systematicness and consistency to learn nutrition knowledge (47). Furthermore, compared with Han students, minority tended to report a low possibility of a high level of NL and three sub-domains, which may be due to poor educational opportunity and access to nutrition knowledge in minority, and some of their customs may lead to incorrect or unhealthy eating behavior (48). With regard to the BMI, previous reports showed an insignificant association between NL and BMI (49, 50), but in our study, students with normal BMI were more likely to be a high level of NL and FNL than students with an abnormal BMI. Nutrition literacy is an important influencing factor of obesity (51). Higher NL is associated with healthy dietary habit and lifestyle (52), and nutrition knowledge plays an essential role in the development and prevention of obesity in later life (53). Meanwhile, given the lack of proper awareness of their own body shape, obese children tend to make unhealthy food options (54). Increasing nutrition knowledge and skills through improving NL and promoting application of nutrition knowledge for healthy eating practices can reduce malnutrition in children (55, 56).

With regard to the school environment, students who were boarding in school, in rural areas and receiving school meal support from the government should be the main target of nutrition education and improvement. Compared with boarders, non-boarding students were exposed to an environment of family that enabled them to engage in nutrition activities and interactions with their caregivers (38). In Michou et al.'s study (18), socioeconomic inequalities were negatively associated with NL. In this study, rural students were less likely to report a high level of NL/FNL/CNL than urban students, probably because rural students were affected by underdeveloped economy, poor basic life implementation, limited access to nutrition information and low awareness of developing good eating behavior (57), which indicated the importance of narrowing the gap between urban and rural nutrition services. Although the Nutrition Improvement Programme for Rural Compulsory Education Students (NIPRCES) has been implemented by the government for 9 years in China (58), students receiving school meal support from the government were still less likely to be a high level of NL and three sub-domains because the shortage of dining facilities and lack of nutrition education was common in NIPRCES districts (58). In addition, current nutrition improvement programme relatively neglected education and practice of nutrition for students (59). Students receiving school meal support from the government districts needed more opportunities to learn and apply the nutrition knowledge and skills to improve their NL.

With regard to family, when the level of education of parents was high, students were more likely to be a high level of NL. Parents with high educational attainment are better equipped to teach children nutrition skills, and they have more resources to create chances for children to learn and practice these skills (60). Increasing NL of children with parents having a low level of education through interventions is critical. In addition to the influence of parents, this study found that primary caregiver was a contributing factor. Children are dependent on their parents with regard to access to material, financial and social resources (61), and some studies have shown that child care of grandparent might increase the risk of children obesity (62). Students with parents' parenting were more likely to be a high level of NL/FNL/CNL than students with other caregivers' parenting. Therefore, measures should be considered to ensure that left-behind children had regular access to nutrition resources (63). Left-behind children are a special group in China, referring to rural children with one or both parents working in urban areas for at least 6 months (64). According to the Ministry of Civil Affairs, rural China had more than six million left-behind children (65). The government of Chongqing has presented the importance of addressing the problems of NL amongst left-behind children by deepening family education support services and popularizing nutrition knowledge (66).

This study was the first cross-sectional study with a large-scale sample targeting NL of middle school students in Chongqing, China. However, the current study had several limitations that should be acknowledged. Although quality control was strictly implemented in the process, online and self-reported survey may bring some information bias inevitably. Height and weight were self-reported by the students. Although schools organize routine health check as required by the government, the gap between these checks and the study varied between schools. Moreover, on-site investigation using CM-NLS can be conducted to evaluate children's NL in a more diverse sample across the entire China in future studies.

## Conclusions

This study showed that the NL of middle school students in Chongqing was at the moderate level and highlighted a low probability of a high level of NL for boys, minority and students who were in senior high school, boarding in school, in rural areas, receiving school meal support from the government, with parents of low education, left behind and with an abnormal BMI. These findings stressed on NL levels of students with different backgrounds, especially with different family economic status and family resources, which recommended health practitioners and policy makers to formulate strategies increasing NL in different groups of students and controlling the impact caused by disparities of family economic and nutrition resources.

## Data Availability Statement

The raw data supporting the conclusions of this article will be made available by the authors, without undue reservation.

## Ethics Statement

The studies involving human participants were reviewed and approved by The Ethics Committee of Chongqing Medical University. Written informed consent to participate in this study was provided by the participants' legal guardian/next of kin.

## Author Contributions

MZ and YZ contributed to conception and design of the study and performed the statistical analysis. MZ organized the database. YZ, ZC, and JX wrote the first draft of the manuscript. SL, TW, ZS, and MS wrote sections of the manuscript. All authors contributed to manuscript revision, read, and approved the submitted version.

## Funding

This work was supported by the Chinese Nutrition Society (Science Popularization and Communication Research Fund project, award Number CNS-SCP2020-34).

## Conflict of Interest

The authors declare that the research was conducted in the absence of any commercial or financial relationships that could be construed as a potential conflict of interest.

## Publisher's Note

All claims expressed in this article are solely those of the authors and do not necessarily represent those of their affiliated organizations, or those of the publisher, the editors and the reviewers. Any product that may be evaluated in this article, or claim that may be made by its manufacturer, is not guaranteed or endorsed by the publisher.

## References

[1] Kalkan I. The impact of nutrition literacy on the food habits among young adults in Turkey. Nutr Res Pract. (2019) 13:352–7. 10.4162/nrp.2019.13.4.352 31388412 PMC6669071

[2] Kazemi A Zahraei NN Nazarian N. The relation between intra- and interpersonal factors and food consumption level among Iranian adolescent girls. Iran J Nurs Midwifery Res. (2016) 21:147–52. 10.4103/1735-9066.178235 27095987 PMC4815369

[3] Shi X Tubb L Fingers ST Chen S Caffrey JL. Associations of physical activity and dietary behaviors with children's health and academic problems. J Sch Health. (2013) 83:1–7. 10.1111/j.1746-1561.2012.00740.x 23253284

[4] United Nations International Children's Emergency Fund,. The State of the World's Children 2019. Children, Food Nutrition: Growing Well in a Changing World. (2019). Available online at: https://www.unicef.org/reports/state-of-worlds-children-2019 (accessed October 14, 2019).

[5] Borges CA Marchioni DML Levy RB Slater B. Dietary patterns associated with overweight among Brazilian adolescents. Appetite. (2018) 123:402–9. 10.1016/j.appet.2018.01.001 29355584

[6] World Health Organization. Global Action to Accelerate Adolescent Health (AA-HA!) Guidance to support national implementation-Summary. (2017). Available online at: https://apps.who.int/iris/bitstream/handle/10665/255418/WHO-FWC-MCA-17.05-chi.pdf?sequence=5 (accessed October 6, 2017).

[7] Pietrobelli A Pecoraro L Ferruzzi A Heo M Faith M Zoller T. Effects of COVID-19 lockdown on lifestyle behaviors in children with obesity living in Verona, Italy: a longitudinal study. Obesity. (2020) 28:1382–5. 10.1002/oby.22861 32352652 PMC7267384

[8] Clemmensen C Petersen MB Sørensen TIA. Will the COVID-19 pandemic worsen the obesity epidemic? Nat Rev Endocrinol. (2020) 16:469–70. 10.1038/s41574-020-0387-z 32641837 PMC7342551

[9] Gibbs HD Ellerbeck EF Befort C Gajewski B Kennett AR Yu Q. Measuring nutrition literacy in breast cancer patients: development of a novel instrument. J Cancer Educ. (2016) 31:493–9. 10.1007/s13187-015-0851-y 25952941 PMC4639469

[10] Silk KJ Sherry J Winn B Keesecker N Horodynski MA Sayir A. Increasing nutrition literacy: testing the effectiveness of print, web site, and game modalities. J Nutr Educ Behav. (2008) 40:3–10. 10.1016/j.jneb.2007.08.012 18174098

[11] Blitstein JL Evans WD. Use of nutrition facts panels among adults who make household food purchasing decisions. J Nutr Educ Behav. (2006) 38:360–4. 10.1016/j.jneb.2006.02.009 17142192

[12] Watson WL Chapman K King L Kelly B Hughes C Yu Louie JC. How well do Australian shoppers understand energy terms on food labels? Public Health Nutr. (2013) 16:409–17. 10.1017/S1368980012000900 22464021 PMC10271533

[13] Krause C Sommerhalder K Beer-Borst S Abel T. Just a subtle difference? Findings from a systematic review on definitions of nutrition literacy and food literacy. Health Promot Int. (2018) 33:378–89. 10.1093/heapro/daw084 27803197 PMC6005107

[14] Ndahura NB,. Nutrition Literacy Status of Adolescent Students in Kampala District, Uganda. (2012). Available online at: https://hdl.handle.net/10642/1200 (accessed July 25, 2012).

[15] Guttersrud Ø Petterson KS. Young adolescents' engagement in dietary behaviour - the impact of gender, socio-economic status, self-efficacy and scientific literacy. Methodological aspects of constructing measures in nutrition literacy research using the Rasch model. Public Health Nutr. (2015) 18:2565–74. 10.1017/S1368980014003152 25634262 PMC10271695

[16] Doustmohammadian A Keshavarz Mohammadi N Omidvar N Amini M Abdollahi M Eini-Zinab H. Food and nutrition literacy (FNLIT) and its predictors in primary schoolchildren in Iran. Health Promot Int. (2019) 34:1002–13. 10.1093/heapro/day050 30101341

[17] Yuen E Y N Thomson M Gardiner H. Measuring nutrition and food literacy in adults: a systematic review and appraisal of existing measurement tools. Health Lit Res Pract. (2018) 2:e134–60. 10.3928/24748307-20180625-01 31294289 PMC6607839

[18] Michou M Panagiotakos DB Lionis C Costarelli V. Socioeconomic inequalities in relation to health and nutrition literacy in Greece. Int J Food Sci Nutr. (2019) 70:1007–13. 10.1080/09637486.2019.1593951 30935258

[19] Gibbs HD Kennett AR Kerling EH Yu Q Gajewski B Ptomey LT. Assessing the nutrition literacy of parents and its relationship with child diet quality. J Nutr Educ Behav. (2016) 48:505–9.e1. 10.1016/j.jneb.2016.04.006 27216751 PMC4931947

[20] Du W Fu J Su C Zhang Q Zhai F Zhang B. Surveys on the nutrition literacy of 802 adults in Jiangxi province. J Hyg Res. (2010) 39:735–8. 10.19813/j.cnki.weishengyanjiu.2010.06.023 21351643

[21] PatelPPanaichSSteinbergJZalawadiyaSKumarAAranhaA. Use of nutrition literacy scale in elderly minority population. J Nutr Health Aging. (2013) 17:894–7. 10.1007/s12603-013-0355-624257573

[22] Aihara Y Minai J. Barriers and catalysts of nutrition literacy among elderly Japanese People. Health Promot Int. (2011) 26:421–31. 10.1093/heapro/dar005 21307024

[23] Cao L Xu J Wang F Huang S Shen L Zhang X. Surveys on the nutrition literacy of rural children in Xuzhou. Jiangsu J Prev Med. (2016) 27:114–16. 10.13668/j.issn.1006-9070.2016.01.044

[24] Harley A Lemke M Brazauskas R Carnegie NB Bokowy L Kingery L. Youth chef academy: pilot results from a plant-based culinary and nutrition literacy program for sixth and seventh graders. J Sch Health. (2018) 88:893–902. 10.1111/josh.12703 30392187 PMC6546111

[25] Ma X Wang J Zhao J Xiao G Wang Y Li J. Analysis of nutrition literacy and lunch behavior of primary and secondary school students in Fengtai District of Beijing in 2018. Occup Health. (2019) 35:2918–22. 10.13329/j.cnki.zyyjk.2019.0743

[26] Liu T Su X Li N Zhu Y Ma G Zhu W. Delphi method on food and nutrition literacy core components for school-age children. Chin J Health Educ. (2020) 36:125–8, 157. 10.16168/j.cnki.issn.1002-9982.2020.02.007

[27] Cassar AM Denyer GS O'Connor HT Gifford JA. A Qualitative investigation to underpin the development of an electronic tool to assess nutrition literacy in Australians adults. Nutrients. (2018) 10:251. 10.3390/nu10020251 29473889 PMC5852827

[28] Neuhauser L Rothschild R Rodríguez FM. MyPyramid.gov: assessment of literacy, cultural and linguistic factors in the USDA food pyramid web site. J Nutr Educ Behav. (2007) 39:219–25. 10.1016/j.jneb.2007.03.005 17606248

[29] Zoellner J You W Connell C Smith-Ray RL Allen K Tucker KL. Health literacy is associated with healthy eating index scores and sugar-sweetened beverage intake: findings from the rural Lower Mississippi Delta. J Am Diet Assoc. (2011) 111:1012–20. 10.1016/j.jada.2011.04.010 21703379 PMC3160591

[30] Persoskie A Hennessy E Nelson WL. US consumers' understanding of nutrition labels in 2013: the importance of health literacy. Prev Chronic Dis. (2017) 14:E86. 10.5888/pcd14.170066 28957033 PMC5621522

[31] Ma G. Dietary guidelines for school-age children. Chin J Sch Health. (2016) 37:961–3, 967. 10.16835/j.cnki.1000-9817.2016.07.001

[32] Yan C. Investigation on Physical Fitness and Nutrition Status of Primary and Middle School Students in Different Regions of Chongqing City. Chongqing: The Third Military Medical University (2013).

[33] Nutbeam D. Health literacy as a public health goal: a challenge for contemporary health education and communication strategies into the 21st century. Health Promot Int. (2000) 15:259–67. 10.1093/heapro/15.3.259

[34] Zeng M. Development and Application of Nutrition Literacy Questionnaire for Middle School Students in Chongqing. Chongqing: Chongqing Medical University (2021).

[35] Cole TJ Bellizzi MC Flegal KM Dietz WH. Establishing a standard definition for child overweight and obesity worldwide: international survey. BMJ. (2000) 320:1240–3. 10.1136/bmj.320.7244.1240 10797032 PMC27365

[36] Iddir M Brito A Dingeo G Fernandez Del Campo SS Samouda H La Frano MR. Strengthening the immune system and reducing inflammation and oxidative stress through diet and nutrition: considerations during the COVID-19 crisis. Nutrients. (2020) 12:1562. 10.3390/nu12061562 32471251 PMC7352291

[37] D'amato-Kubiet L. Nutrition Literacy and Demographic Variables as Predictors of Adolescent Weight Status in a Florida County. Orlando, FL: University of Central Florida (2013).

[38] Liu T Su X Li N Sun J Ma G Zhu W. Development and validation of a food and nutrition literacy questionnaire for Chinese school-age children. PLoS ONE. (2021) 16:e0244197. 10.1371/journal.pone.0244197 33406105 PMC7787443

[39] Austin EW Austin BW French BF Cohen MA. The effects of a nutrition media literacy intervention on parents' and youths' communication about food. J Health Commun. (2018) 23:190–9. 10.1080/10810730.2018.1423649 29338585

[40] Law QPS Yau AHY Chung JWY. Chinese adults' nutrition label literacy in Hong Kong: implications for nurses. Nurs Health Sci. (2019) 21:171–7. 10.1111/nhs.12575 30345724

[41] Liu P Yu Y King L Li M. Snack and beverage consumption and preferences in a sample of Chinese children - are they influenced by advertising? Asia Pac J Clin Nutr. (2017) 26:1125–32. 10.6133/apjcn.012017.04 28917240

[42] Norman J Kelly B McMahon AT Boyland E Baur LA Chapman K. Sustained impact of energy-dense TV and online food advertising on children's dietary intake: a within-subject, randomised, crossover, counter-balanced trial. Int J Behav Nutr Phys Act. (2018) 15:37. 10.1186/s12966-018-0672-6 29650023 PMC5897936

[43] Amin SA Panzarella C Lehnerd M Cash SB Economos CD Sacheck JM. Identifying food literacy educational opportunities for youth. Health Educ Behav. (2018) 45:918–25. 10.1177/1090198118775485 29848139

[44] Lanpher MG Askew S Bennett GG. Health literacy and weight change in a digital health intervention for women: a randomized controlled trial in primary care practice. J Health Commun. (2016) 21(Suppl. 1):34–42. 10.1080/10810730.2015.1131773 27043756 PMC4935541

[45] Wu JR Moser DK DeWalt DA Rayens MK Dracup K. Health literacy mediates the relationship between age and health outcomes in patients with heart failure. Circ Heart Fail. (2016) 9:e002250. 10.1161/CIRCHEARTFAILURE.115.002250 26721913 PMC4698978

[46] Vaitkeviciute R Ball LE Harris N. The relationship between food literacy and dietary intake in adolescents: a systematic review. Public Health Nutr. (2015) 18:649–58. 10.1017/S1368980014000962 24844778 PMC10271388

[47] Zhang Z Zhu R Chai W. Current condition and its influencing factors' analysis on nutrition knowledge of middle school students in Beijing. Food Nutr China. (2020) 26:77–81. 10.19870/j.cnki.11-3716/ts.2020.01.019

[48] Abdel-Latif MMM Saad SY. Health literacy among Saudi population: a cross-sectional study. Health Promot Int. (2019) 34:60–70. 10.1093/heapro/dax043 28973389

[49] Sichert-Hellert W Beghin L De Henauw S Grammatikaki E Hallström L Manios Y. Nutritional knowledge in European adolescents: results from the HELENA (Healthy Lifestyle in Europe by Nutrition in Adolescence) study. Public Health Nutr. (2011) 14:2083–91. 10.1017/S1368980011001352 21810282

[50] Kalkan I Turkmen AS Filiz E. Dietary habits of Turkish adolescents in Konya, Turkey. New Trends Issues Proc Adv Pure Appl Sci. (2016) 7:190–96. 10.18844/gjpaas.v0i7.317934857207

[51] Joulaei H Keshani P Kaveh M H. Nutrition literacy as a determinant for diet quality amongst young adolescents: a cross sectional study. Prog Nutr. (2018) 20:455–64. 10.23751/pn.v20i3.6705

[52] Jones C L Adkins K. Nutrition literacy, food preference, and food choices within a school-based choice food pantry. J Hunger Environ Nut. (2021) 16:1–17. 10.1080/19320248.2021.1873882

[53] Hruby A Hu FB. The epidemiology of obesity: a big picture. Pharmacoeconomics. (2015) 33:673–89. 10.1007/s40273-014-0243-x 25471927 PMC4859313

[54] Liu H Liu Z Reyila T Yang Y Sun Y Lin Y. Investigation on the relationship between overweight and obesity and KAP among middle school students in Fangshan District of Beijing. Occup Health. (2020) 36:1402–6. 10.13329/j.cnki.zyyjk.2020.0372

[55] Koca B Arkan G. The relationship between adolescents' nutrition literacy and food habits, and affecting factors. Public Health Nutr. (2020) 24:1–12. 10.1017/S1368980020001494 32723409 PMC11574834

[56] Taleb S Itani L. Nutrition literacy among adolescents and its association with eating habits and BMI in Tripoli, Lebanon. Diseases. (2021) 9:25. 10.3390/diseases9020025 33805571 PMC8103266

[57] Wang W Zhang Y Lin B Mei Y Ping Z Zhang Z. The urban-rural disparity in the status and risk factors of health literacy: a cross-sectional survey in central China. Int J Environ Res Public Health. (2020) 17:3848. 10.3390/ijerph17113848 32485790 PMC7312746

[58] Zhang F Hu X Tian Z Zhang Q Ma G. Literature research of the Nutrition Improvement Programme for Rural Compulsory Education Students in China. Public Health Nutr. (2015) 18:936–43. 10.1017/S1368980014001001 24866472 PMC10271158

[59] Wang Y Gao L Thilaphong S Zhang X Xu F Wang S. Investigation on nutrition improvement of school-age children in cross-border region in Yunnan province. Soft Sci Health. (2018) 32:28–31, 38. 10.3969/j.issn.1003-2800.2018.01.008

[60] Fleary SA Joseph P Pappagianopoulos JE. Adolescent health literacy and health behaviors: a systematic review. J Adolesc. (2018) 62:116–27. 10.1016/j.adolescence.2017.11.010 29179126

[61] Bröder J Okan O Bauer U Bruland D Schlupp S Bollweg TM. Health literacy in childhood and youth: a systematic review of definitions and models. BMC Public Health. (2017) 17:361. 10.1186/s12889-017-4267-y 28441934 PMC5405535

[62] An R Xiang X Xu N Shen J. Influence of grandparental child care on childhood obesity: a systematic review and meta-analysis. Childhood Obes. (2020) 16:141–53. 10.1089/chi.2019.0246 31971822

[63] Fellmeth G Rose-Clarke K Zhao C Busert LK Zheng Y Massazza A. Health impacts of parental migration on left-behind children and adolescents: a systematic review and meta-analysis. Lancet. (2018) 392:2567–82. 10.1016/S0140-6736(18)32558-3 30528471 PMC6294734

[64] Wen YJ Hou WP Zheng W Zhao XX Wang XQ Bo QJ. The neglect of left-behind children in China: a meta-analysis. Trauma Viol Abuse. (2020) 22:1326–38. 10.1177/1524838020916837 32308147

[65] Ministry of Civil Affairs of the People's Republic of China. Ministry of Civil Affairs' Reply to ‘Suggestions on Further Strengthening Mental Health Education of Left-behind Children in Rural Areas'. (2019). Available online at: http://xxgk.mca.gov.cn:8011/gdnps/pc/content.jsp?id=12518&mtype=4 (accessed December 13, 2019).

[66] Civil Affairs Bureau of Chongqing. Opinions on Further Improving the Care and Service System for Left-Behind Children and Children in Difficulties in Rural Areas. (2020). Available online at: http://mzj.cq.gov.cn/zwgk_218/fdzdgknr/zcwj/xzgfxwj/bdwwj/202010/t20201026_8094142.html (accessed October 10, 2020).

