# Supplementary material for: Nutrition Literacy of Middle School Students and Its Influencing Factors: A Cross-Sectional Study in Chongqing, China
Source: Front Public Health. 2022 Mar 15;10:807526. doi: 10.3389/fpubh.2022.807526 (PMC8965039; doi:10.3389/fpubh.2022.807526)
Supplement: Supplementary file 1 [file Data_Sheet_1.PDF]

Section B of ‘CM-NLS’

	**Scale items /Question**	**option**
	**1. Functional NL**	
	1.1 obtain	
Q1_1.1.1	I will seek answers when I don't know anything about nutrition. **[liket-5]**	①Strongly disagree ②Disagree ③Neutral ④Agree ⑤Strongly agree
Q2_1.1.2	Know where to find accurate information when I have a nutrition-related problem or want to learn healthy eating behaviors. **[liket-5]**	①Strongly disagree ②Disagree ③Neutral ④Agree ⑤Strongly agree
Q3_1.1.3	It is not difficult for me to find the need nutritional information from a large number of information sources. **[liket-5]**	①Strongly disagree ②Disagree ③Neutral ④Agree ⑤Strongly agree
	1.2 understand	
Q4_1.2.1	Learn about food sources and categories **[sorting problem]**	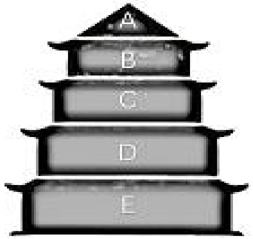 ①Pork, chicken, duck, fish, eggs, etc.<br>②Rice noodles, sweet potatoes, mung beans, etc.<br>③Milk and milk products, soybeans and their products<br>④Edible oil, salt<br>⑤Vegetables and fruits
Q5_1.2.2	Whole grains (millet, corn, etc.) are more	① True ②False ③Don’t know

	nutritious than refined grains (rice, flour, etc.). **T**	
Q6_1.2.3	Know the advantage that eats bean curd, soya-bean milk to wait for soybean product**[multi-select]**	①Good for health ②Good for patients with cardiovascular disease ③Increase the intake of high-quality protein ④ Prevent the adverse effects of excessive consumption of meat ⑤Don't know
Q7_1.2.4	Smoking and salting foods can increase the risk of cancer. **T**	① True ②False ③Don't know
Q8_1.2.5	Being overweight or underweight increases the risk of disease. **T**	① True ②False ③Don't know
Q9_1.2.6	When eating meat, try to eat lean meat, fat meat can eat, but cannot eat more. **T**	① True ②False ③Don't know
Q10_1.2.7	Good eating habits can prevent chronic diseases such as hypertension and diabetes. **T**	① True ②False ③Don't know
Q11_1.2.8	You can reduce your exercise if you eat less. **F**	① True ②False ③Don't know
Q12_1.2.9	Drink water in small quantities more than once. **T**	① True ②False ③Don't know
Q13_1.2.10	Separate meals can help prevent mouth to mouth diseases. **T**	① True ②False ③Don't know
Q14_1.2.11	It is easy to understand the contents of the Dietary Guidelines for Chinese residents. **[liket-5]**	①Never heard ②Strongly disagree ③Disagree ④Neutral ⑤Agree ⑥Strongly agree
Q15_1.2.12	It is easy to understand the nutritional information (such as energy, protein, sugar, etc.) on food packaging. **[liket-5]**	① Strongly disagree ②Disagree ③Neutral ④Agree ⑤Strongly agree
Q16_1.2.13	It is easy to understand that recommendations relating to health and nutrition in secondary school students. **[liket-5]**	① Strongly disagree ②Disagree ③Neutral ④Agree ⑤Strongly agree

Q17_1.2.14	It is easy to understand nutrition information you read in a brochure, book or on the Internet. **[liket-5]**	① Strongly disagree ②Disagree ③Neutral ④Agree ⑤Strongly agree
	1.3 apply/use	
Q18_1.3.1	When eating, consider **nutrition** first. **[single-select]**	① Taste ②Nutrition ③Satisfy hunger ④Maintain body ⑤Other ⑥No standard
Q19_1.3.2	Use **nutrition labels** to choose foods wisely. **[multi-select]**	①Production date ②expiration date ③Nutrient content table ④Manufacturer ⑤ Don't know ⑥I haven't paid attention to it
Q20_1.3.3	Choose snacks wisely. **[multi-select]**	This option can be modified according to the food culture habits of each country.
Q21_1.3.4	Don't substitute fruits for vegetables. **[liket-5]**	① Never experienced ②Never ③Occasionally ④Sometimes ⑤Always
Q22_1.3.5	Don't replace fresh vegetables with pickles and pickles. **[liket-5]**	① Never experienced ②Never ③Occasionally ④Sometimes ⑤Always
Q23_1.3.6	Don't snack instead of meals. **[liket-5]**	① Never experienced ②Never ③Occasionally ④Sometimes ⑤Always
Q24_1.3.7	Do not use canned fruit, preserved fruit and other processed fruit products instead of fresh fruit. **[liket-5]**	① Never experienced ②Never ③Occasionally ④Sometimes ⑤Always
Q25_1.3.8	Cooked food should be kept at room temperature for no more than 2 hours. **[liket-5]**	① Never experienced ②Never ③Occasionally ④Sometimes ⑤Always
Q26_1.3.9	Eat a variety of foods: eat at least 12 foods a day and 25 foods a week. **[single-select]**	Give an example, ask the respondent to fill in the type of food consumed in the past 24 hours ①0~4 ②5~8 ③9~11 ④≥12 ⑤Don't know
Q27_1.3.10	Eat breakfast every day. **[single-select]**	The frequency of eating breakfast in the past week①0/weekly ②1~2/weekly ③ 3~4/weekly ④5~6/weekly ⑤Every day
Q28_1.3.11	Eat fruit every day. **[single-select]**	The frequency of eating fresh fruit in the past week①0/weekly ②1/weekly ③2~6 /weekly ④1 /day ⑤≥2 /day
Q29_1.3.12	Drink milk every day. **[single-select]**	The frequency of drinking milk in the past week①0/weekly ②1/weekly ③2~6

		/weekly ④1 /day ⑤≥2 /day
Q30_1.3.13	Avoid or limit sugary drinks. **[single-select]**	The frequency of drinking sugary drinks in the past week①0/weekly ②1/weekly ③2~6 /weekly ④1 /day ⑤≥2 /day
Q31_1.3.14	Don't drink. **[single-select]**	① Yes ②No
Q32_1.3.15	Cut down on Western fast food. **[single-select]**	The frequency of eating Western fast food in the past week① 0/weekly ② 1/weekly ③2~6 /weekly ④1 /day ⑤≥2 /day
Q33_1.3.16	Cut down on fat, smoke and spicy foods. **[single-select]**	The frequency of eating fat, smoke and spicy foods in the past week① 0/weekly ②1/weekly ③2~6 /weekly ④1 /day ⑤≥2 /day
Q34_1.3.17	Do not patronize food and drink stalls such as roadside stands. **[liket-5]**	① Never experienced ②Never ③Occasionally ④Sometimes ⑤Always
Q35_1.3.18	Knowledge of healthy weight and can rightly realize body shape. **[single-select]**	Respondents were asked to evaluate their body shape after self-reporting their height(m) and weight(kg) ① Thin ② Normal ③ Overweight ④ Obese ⑤ Don't know
	**2. Interactive NL**	
	2.1 interact	
Q36_2.1.1	An active attitude toward obtaining nutritional information. **[liket-5]**	① Never experienced ②Never ③Occasionally ④Sometimes ⑤Always
Q37_2.1.2	The willingness communicate to learn about nutrition and health. **[liket-5]**	① Strongly disagree ②Disagree ③Neutral ④Agree ⑤Strongly agree
Q38_2.1.3	The willingness to receive nutrition education. **[liket-5]**	① Strongly disagree ②Disagree ③Neutral ④Agree ⑤Strongly agree
Q39_2.1.4	The willingness to change poor eating habits using the nutrition knowledge learned. **[liket-5]**	① Strongly disagree ②Disagree ③Neutral ④Agree ⑤Strongly agree
Q40_2.1.5	The willingness to persuade others to change their bad eating habits. **[liket-5]**	① Strongly disagree ②Disagree ③Neutral ④Agree ⑤Strongly agree

	**3. Critical NL**	
	3.1 medial literacy(ML)	
Q41_3.1.1	Talk about nutrition with others (e.g. friends, family, etc.). **[liket-5]**	① Strongly disagree ②Disagree ③Neutral ④Agree ⑤Strongly agree
Q42_3.1.2	Take the initiative to disseminate nutrition knowledge to others. **[liket-5]**	①Never experienced ②Never ③seldom ④Occasionally ⑤Sometimes ⑥Always
Q43_3.1.3	Attention to nutritional information in the media. **[liket-5]**	①Never experienced ②Never ③seldom ④Occasionally ⑤Sometimes ⑥Always
Q44_3.1.4	Critical of nutrition information from all sources in society. **[liket-5]**	① Never experienced ② Never ③ seldom ④ Occasionally ⑤ Sometimes ⑥ Always
Q45_3.1.5	I can judge the accuracy and scientific nature of nutrition-related information in the media. **[liket-5]**	① Never experienced ② Never ③ seldom ④ Occasionally ⑤ Sometimes ⑥ Always
Q46_3.1.6	When confronted with a great amount of nutritional information, I can judge whether the information is right or wrong through media reports. **[liket-5]**	① Never experienced ② Never ③ seldom ④ Occasionally ⑤ Sometimes ⑥ Always
Q47_3.1.7	The extent to which nutritional information in the media affects you. **[liket-5]**	① Never experienced ② Never ③ seldom ④ Occasionally ⑤ Sometimes ⑥ Always
Q48_3.1.8	Dare to question deeply rooted social and cultural phenomena related to food and health. **[liket-5]**	① Never experienced ② Never ③ seldom ④ Occasionally ⑤ Sometimes ⑥ Always
	3.2 critical	
Q49_3.2.1	How easy it is to distinguish whether nutritional information is scientific or not. **[liket-5]**	①Never experienced ②Very difficult ③Slightly difficult ④General ⑤Slightly simple ⑥Very simple
Q50_3.2.2	How easy it is to distinguish between healthy and	①Never experienced ②Very difficult ③Slightly difficult ④General ⑤Slightly

	less healthy foods. **[liket-5]**	simple ⑥Very simple
Q51_3.2.3	How easy it is to assess the impact of eating habits on health. **[liket-5]**	①Never experienced ②Very difficult ③Slightly difficult ④General ⑤Slightly simple ⑥Very simple
Q52_3.2.4	When I am given advice on healthy eating, I can judge what fits my health needs. **[liket-5]**	①Never experienced ②Very difficult ③Slightly difficult ④General ⑤Slightly simple ⑥Very simple
